# A new *Agkistrodon halys* venom-purified protein C activator prevents myocardial fibrosis in diabetic rats

**DOI:** 10.3325/cmj.2015.56.439

**Published:** 2015-10

**Authors:** Shu Li, Yun Hong, Xin Jin, Genbao Zhang, Zaichang Hu, Liuwang Nie

**Affiliations:** 1 Life Science College, Anhui Normal University, Wuhu, China; 2 Department of Pathophysiology, Wannan Medical College, Wuhu, China; 3 Department of Ultrasonography, Yijishan Hospital, Wuhu, China; 4 Department of Pharmacology, Wannan Medical College, Wuhu, China; 5 Department of Clinic Medicine, Wannan Medical College, Wuhu, China; The first two authors contributed equally to the study.

## Abstract

**Aim:**

To assess the effects of protein C activator (PCA) from *Agkistrodon halys* snake venom on cardiac fibrosis in streptozotocin (STZ) induced diabetic rat model, and investigate the mechanisms of its action.

**Methods:**

PCA was identified by one-dimensional reversed phase liquid chromatography – mass spectrometry/mass spectrometry. Male Sprague-Dawley rats (120-140 g) were randomly assigned to negative control (NC) and diabetic group. Diabetes was induced by STZ in high-fat diet fed rats. Diabetic group was subdivided into three groups: diabetic group (DM), diabetic group treated with PCA (0.5, 2, and 8 mg/kg), and diabetic group treated with metformin (5 mg/kg, positive control). NC and DM groups received the same volume of distilled water. Left ventricular mass index (LVWI) and collagen volume fraction were measured by hematoxylin and eosin and Masson staining. Transforming growth factor beta-1 (TGF-β1) and interleukin 1 beta (IL-1β) levels were determined by enzyme-linked immunosorbent assay.

**Results:**

The diabetic rat model was successfully established by STZ induction and high-fat diet. Glucose level, LVWI, TGF-β1 and IL-1β level, and collagen volume fraction were significantly reduced in diabetic rats treated by PCA in a dose-dependent manner (*P* < 0.050), especially in the high dose (8 mg/kg) group (*P* < 0.010), compared to diabetes group. The high dose PCA had the same effect as metformin positive control in reducing the level of fasting blood glucose. PCA decreased the expression of MMP-2 and reduced that of TIMP-2.

**Conclusion:**

Our results indicate that PCA has anti-fibrotic effects and that it may be used to treat myocardial fibrosis.

The main complications and leading causes of death among patients with diabetes mellitus are coronary artery disease (CAD), hypertension, and diabetic cardiomyopathy (DCM), a direct adverse effect of diabetes on the heart (1). Epidemiological data showed that the risk for heart failure is respectively 2.4-fold and 5-fold higher in diabetic men and women than in non-diabetic individuals (2). Indeed, diabetes is a significant risk factor for heart failure and an independent risk factor for increased mortality among individuals with heart failure (3). Myocardial fibrosis (MF) plays a major role in the pathophysiology of DCM (4). It is also strongly associated with some angiocardiopathy diseases such as hypertension, DCM, rheumatic heart disease, and myocardial infarction (5,6).

Fibrosis is a complex process characterized by cardiac fibroblast accumulation and excess extracellular matrix deposition of collagen I and III (7), which is a leading cause of diabetes and heart failure. Therefore, in diabetes and many cardiovascular diseases, it is vital to develop appropriate drugs to prevent MF. Angiotensin-converting enzyme inhibitors (ACEI) have been shown in animal models to prevent and treat MF in early stage; clinically, ACEI improve ventricular remodeling and the prognosis of heart failure (8). However, they cause adverse reactions such as cough, hypotension, and nausea, and increase the treatment costs, which all indicates the need for novel candidates in MF treatment.

Snake venom contains multiple proteins and peptides with different structure and function (9), and is an extremely rich source of pharmacologically active molecules with a considerable clinical and medical potential (10-14). Many toxins are being explored and developed for treatment of hypertension, thrombosis, and cancer. In recent years, special attention has been paid to proteins affecting hemostasis, in order to design new therapeutic agents for blood coagulation and other hematological disorders (15). Protein C activators (PCA) are proteases that activate protein C in the mammalian coagulation system *in vitro*. PCA activity was first reported in venom extracts of the snake *Agkistrodon contortrix contortrix* in 1985 (16). The reptilian PCA has attracted increasing attention in both clinical and basic research (17). Other functions of snake venom PCA have to be further studied. Therefore, we hypothesized that snake venom PCA could protect against cardiac fibrosis in DCM. We established a type 2 diabetes model to investigate the cardio-protective properties of PCA in DCM and determine the underlying mechanisms of its action.

## Materials and methods

### PCA purification and liquid chromatography – mass spectrometry/mass spectrometry

The isolation and purification of PCA were carried out as previously described (15). Briefly, DEAE-Cellulose, CM-Sephadex C-50, and SP-Sephadex G-75 (GE, Pittsburgh, PA, USA) column chromatography were used to purify PCA from *Agkistrodon halys* venom. Kaolin partial thromboplastin time, prothrombin time of normal rabbit plasma, and color producing reaction ability (determined by chromogenic substrate assay) were used to assess the anticoagulant activity of PCA-containing fractions. The respective kits were purchased from SUNBIO Co., Ltd (Shanghai, China). The purified PCA was digested by trypsin and analyzed by one-dimensional reversed phase liquid chromatography – mass spectrometry/mass spectrometry (1D LC-MS/MS, Thermo Fisher Scientific, Waltham, MA, USA) at the Research Center for Life Sciences, University of Science and Technology of China in 2012.

### Experiments design

Male Sprague-Dawley (SD) rats (120-140 g) were obtained from the Nanjing Qinlong Shan Animal Experimental Center. They were housed at 22°C under a 12-h light-dark cycle. All experimental procedures were approved by the Animal Care Committee of Wannan Medical College. After one week of adaptation, the animals were randomly assigned to negative control (NC, n = 8) and diabetic (n = 50) group. Control animals received normal chow, and the diabetic group animals were fed a high-fat diet (34.5% fat, 17.5% protein, 48% carbohydrate; HFK Bio-Technology, Beijing, China). Four weeks later, rats were intraperitoneally injected with a single dose of streptozotocin (STZ) (Sigma, St. Louis, MO, USA) at 30 mg/kg dissolved in 0.1 mol/L citrate buffer (pH 4.5) (18). Negative control rats were intraperitoneally injected with citrate buffer alone. One week after STZ injection, rats with fasting blood glucose (FBG) levels exceeding 11.1 mmol/L in two consecutive analyses were considered to be diabetic. Diabetic rats were subdivided into three groups: diabetes group (DM, n = 8), diabetes group treated by gavage with PC (0.5, 2, and 8 mg/kg, n = 24), and diabetes group treated by metformin (5 mg/kg, positive control, n = 8). NC and DM groups received the same volume of distilled water. Ten weeks after the first drug gavage, rats were sacrificed, and the hearts were excised and weighed.

### Tissue preparation

The heart was excised from the chest and weighted after trimming of large vessels. A left ventricular (LV) section was cut off transversely at the mid-ventricular level and fixed in paraformaldehyde (4%). Histological paraffin-embedded sections (5 μm) were prepared for histology staining. The remaining portion of the LV tissue was snap frozen and stored at -80°C for Western blot analysis.

### Hematoxylin and eosin (HE) and Masson staining

Left ventricular tissues were fixed in 4% neutral formaldehyde, paraffin-embedded, and sliced to 5-μm sections. The sections were stained with HE and Masson’s trichrome staining (measure of fibrosis). Collagen volume fraction (CVF) was analyzed with the Image-Pro Plus 5.0 analysis software (Media Cybernetics, Rockville, MD, USA). Perivascular collagen was excluded from the CVF assessment.

### Enzyme-linked immunosorbent assay (ELISA)

Blood samples were centrifuged at 1000 × g for 15 min at 4°C, and plasma samples were immediately stored at -70°C until use. Plasma transforming growth factor beta-1 (TGF-β1) and interleukin 1 beta (IL-1β) levels were measured with commercially available ELISA kits (Jiancheng Bioengineering Institute, Nanjing, China) according to the manufacturer’s instructions. Each sample was analyzed in duplicate.

### RNA isolation and quantitative real-time polymerase chain reaction (qRT-PCR)

Total RNA was isolated from heart tissue samples using TRIzol reagent (Invitrogen, Waltham, MA, USA). Reverse transcription (RT) was performed with the RT reagent kit (Takara Biotechnology Co., Ltd, Dalian, China); qRT PCR (qRT-PCR) was carried out with SYBR Premix Ex *Taq*II (Takara Biotechnology). All reactions were performed according to the manufacturer’s protocols. The following primers were used: matrix metallopeptidase (MMP)-2 (Forward, 5′- CGATGTCTCCCCCAAAACAG -3′; Reverse, 5′- GCATGGTCTCGATGGTGTTC -3′); β-actin (Forward, 5′-CTGCACCACCAACTGCTTAG-3′; Reverse, 5′-AGGTCCACCACTGACACGTT-3′); tissue inhibitor of metalloproteases (TIMP) (Forward, 5′-TCCCCAGAAATCATCGAGAC-3′; Reverse, 5′-ATGGCTGAACAGGGAAACAC-3′). Amplification was carried out on an ABI prism 7300 Real-Time PCR system (Applied Biosystems, Carlsbad, USA). Data were analyzed by the ΔΔCt method (19), with β-actin as an internal control.

### Western blotting

Protein extraction from LV tissues was performed using RIPA buffer (20). A total of 40 μg protein from each sample was separated by 10% SDS-polyacrylamide gel electrophoresis, and electrophoretically transferred to polyvinylidene fluoride membranes. The membranes were blocked with 5% skimmed milk for 1 h, and incubated with specific primary antibodies, including anti-MMP2 (Abcam, Cambridge, UK. 1:2000), anti-TIMP2 (Abcam, 1:1000), and anti-β-actin (Sigma, St. Louis, MO, USA 1:5000) antibodies overnight at 4°C. After incubation with horseradish peroxidase-conjugated secondary antibodies (Abcam) for 2 h, chemiluminescent signals were detected with the EasySee Western Blot Kit (Biyuntian, Hangzhou, China). Band intensity was analyzed using the Adobe Photoshop 7.0.1 software (Adobe Systems Incorporated, San Jose, CA, USA) and compared with the internal standard β-actin.

### Statistical analysis

Experimental data are presented as mean ± standard deviation (SD), since all the values were normally distributed. All statistical analyses were performed using one way analysis of variance (ANOVA) and *t* test for independent samples. *P* < 0.050 was considered statistically significant. Statistical analysis was performed using SPSS v. 19.0 (IBM Corp, Armonk, NY, USA).

## Results

### PCA identification

The mass spectrum of the *Agkistrodon halys* venom purified PCA was compared with *Agkistrodon halys* database in NCBI using the SEQUEST algorithm (Supplementary Table 1(web extra material 1)) (21). Interestingly, this PCA has not been previously reported, but showed 90.27% similarity with acurhagin precursor (*Deinagkistrodon acutus*) (22). These results suggest that PCA is a new metalloproteinase.

### Glucose level, body weight, and left ventricular weight of rats

The STZ induced diabetic rats displayed yellow fur, polydipsia, polyuria, and reduced body weight. Interestingly, PCA affected these symptoms in a dose dependent manner. Diabetic rats had significantly higher glucose level and left ventricular mass index (LVWI) than negative controls (*P* = 0.003) (23). However, glucose level and LVWI in diabetic rats were reduced after treatment with PCA in a dose dependent manner. PCA treatment with 0.5, 2, and 8 mg/kg reduced LVWI to 3.13 ± 0.17, 2.77 ± 0.21, and 2.22 ± 0.23, respectively, and FBG levels to 16.53 ± 1.79, 13.88 ± 1.17, and 10.26 ± 1.09 mmol/L, respectively. In positive control group, LVWI and FBG were also reduced to 2.08 ± 0.19 and 9.37 ± 1.30 mmol/L, respectively. Of note, FBG and LVWI were still higher in the PCA groups than in negative controls. Taken together, these data suggested that the model of diabetes in rats was successfully established, and diabetes symptoms were alleviated by PCA (Table 1).

**Table 1:** Left ventricular mass index (LVWI) and fasting blood glucose (FBG) in negative control group (NC), diabetic group (DM), diabetic groups treated with different doses of protein C activator (PCA), and positive control diabetic group treated with metformin (PC). All values are given as mean ± standard deviation

Group	LVWI (lvw[mg]/bodyweight[g])	FBG (mmol/L)
NC (n = 8)	1.98 ± 0.13	4.93 ± 0.20
DM (n = 8)	3.57 ± 0.24*	20.11 ± 2.61*
DM+ 0.5 mg/kg PCA (n = 8)	3.13 ± 0.17**^†^**	16.53 ± 1.79**^†^**
DM+ 2 mg/kg PCA (n = 8)	2.77 ± 0.21**^†^**	13.88 ± 1.17**^†^**
DM+ 8 mg/kg PCA (n = 8)	2.22 ± 0.23^‡^	10.26 ± 1.09^‡^
PC (n = 8)	2.08 ± 0.19^‡^	9.37 ± 1.30^‡^

*P < 0.001 compared with NC group.

**†***P* < 0.009 compared with DM group.

‡*P* < 0.001 compared with DM group.

### Effects of PCA on plasma TGF-β1 and IL-1β levels

In diabetic rats, circulating TGF-β1 (102.29 ± 16.46 vs 65.98 ± 10.88 pg/mL) and IL-1β (126.08 ± 25.03 vs 82.84 ± 12.34 pg/mL) were higher than in negative control group (*P* = 0.001). However, treatment with middle (2 mg/kg) and high doses (8 mg/kg) of PCA significantly decreased the levels of TGF-β1 (12.23 ± 1.37 and 9.77 ± 1.23 pg/mL) and IL-1β (157.65 ± 10.43 and 124.44 ± 9.86 pg/mL), respectively (*P* = 0.031 vs diabetic group for both). These results indicate that PCA suppressed the expression of TGF-β1 and IL-1β (Table 2).

**Table 2:** Levels of transforming growth factor beta-1 (TGF-β1) and interleukin 1 beta (IL-1β) in in negative control group (NC), diabetic group (DM), diabetic groups treated with different doses of protein C activator (PCA), and positive control diabetic group treated with metformin (PC). All values are given as mean ± standard deviation

Group	TGF-β1 (pg/mL)	IL-1β (pg/mL)
NC (n = 8)	5.63 ± 0.67	97.46 ± 7.48
DM (n = 8)	17.33 ± 2.06^*^	213.79 ± 13.66^*^
DM+ 0.5 mg/kg PCA (n = 8)	14.19 ± 1.86^†^	170.41 ± 12.97^†^
DM+ 2 mg/kg PCA (n = 8)	12.23 ± 1.37^†^	157.65 ± 10.43^†^
DM+ 8 mg/kg PCA (n = 8)	9.77 ± 1.23^‡^	124.44 ± 9.86^‡^
PC (n = 8)	10.31 ± 0.87^‡^	134.26 ± 1.17^‡^

*P = 0.004 compared with NC group.

†*P* = 0.003 compared with DM group.

‡*P* < 0.001 compared with DM group.

### Effects of PCA on cardiac fibrosis

**Figure 1:**
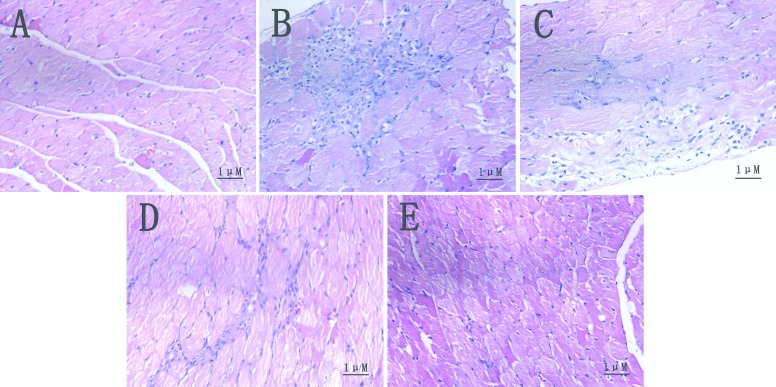
Effect of protein C activator (PCA) on cardiac morphology hematoxylin and eosin staining of sections. (**A**) negative control group (n = 8); (**B**) diabetes model group (n = 8); (**C**) diabetic rats treated with PCA at 0.5 mg/kg (n = 8); (**D**) diabetic rats treated with PCA at 2 mg/kg (n = 8); (**E**) diabetic rats treated with PCA at 8 mg/kg (n = 8). Magnification: ×200.

**Figure 2:**
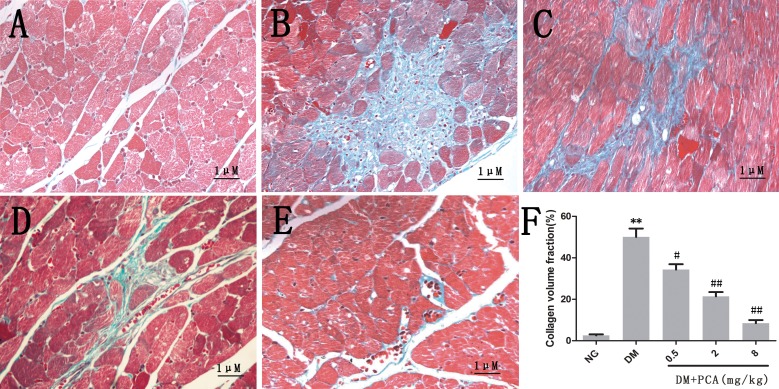
Effect of protein C activator (PCA) on cardiac fibrosis Masson’s trichrome staining: (**A**) negative control group (n = 8); (**B**) diabetes model group (n = 8); (**C**) diabetic rats treated with PCA at 0.5 mg/kg (n = 8); (**D**) diabetic rats treated with PCA at 2 mg/kg (n = 8); (**E**) diabetic rats treated with PCA at 8 mg/kg (n = 8). Positive stained fibrotic areas appear blue. Magnification: ×200.

The effects of PCA on cardiac injury were examined after HE staining. The cardiomyocytes in the negative control group were well-organized, while in DM animals they were disorganized. PCA treatment ameliorated myocyte disorganization (Figure 1). The degree of myocardial fibrosis was assessed by Masson’s trichrome staining (Figure 2). Positively stained fibrotic areas (collagen staining in blue color) were evaluated under a microscope. DM group showed a diffuse, reticular, pockety, and disorganized collagen network. A significant increase in the degree of fibrosis was found in DM rats as compared with negative control animals (*P* < 0.001). However, PCA markedly attenuated these pathological changes (Figure 2). As compared with negative controls, CVF was significantly increased in diabetic rats (Figure 2F). However, in this group PCA treatment overtly decreased CVF values in a dose dependent manner. These results suggested that PCA inhibited the cardiac expression of collagen.

### Effect of PCA on MMP-2 and TIMP-2 mRNA and protein expression levels

**Figure 3:**
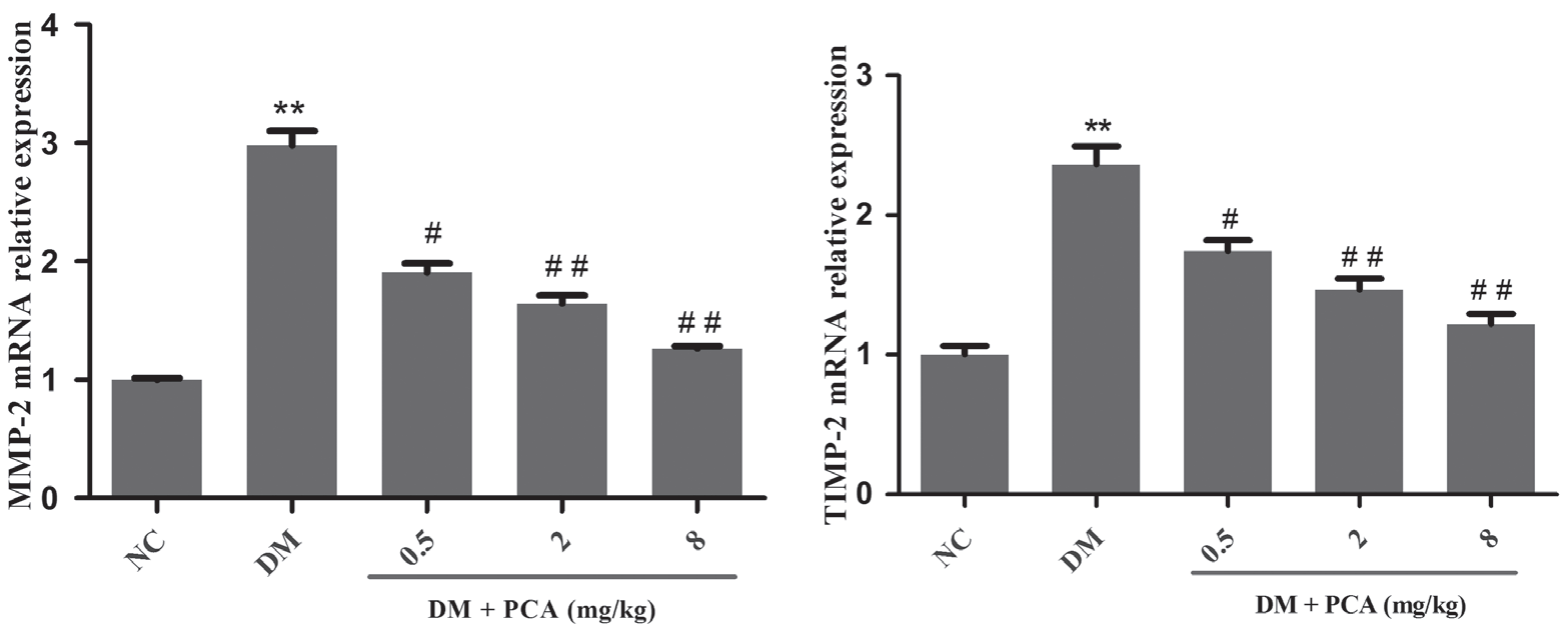
Effect of protein C activator (PCA) on mRNA expression of matrix metallopeptidase-2 and tissue inhibitor of metalloproteases-2. Data are presented as mean ± standard deviation (n = 8); *P < 0.050 and **P < 0.010 compared with negative control group; ^#^*P* < 0.050 and ^##^*P* < 0.010 compared with diabetes mellitus group.

**Figure 4:**
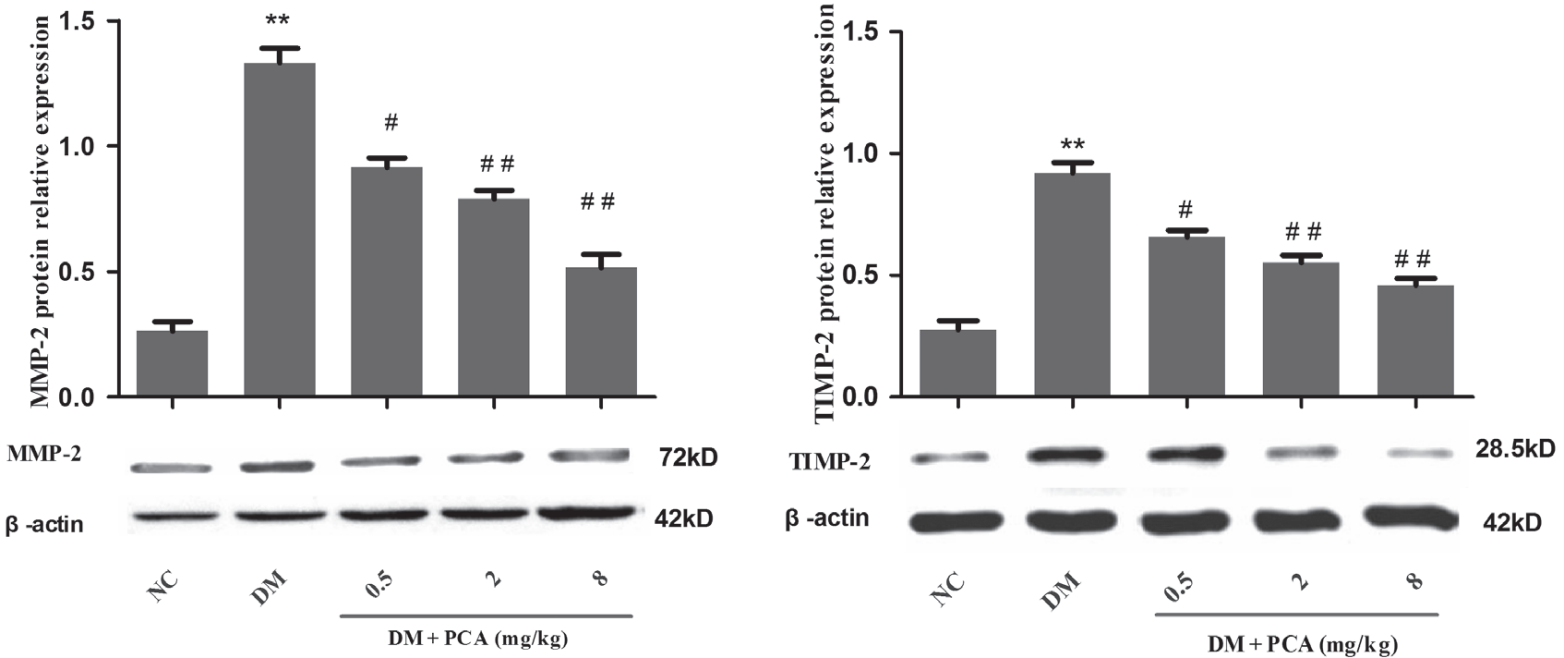
Effects of protein C activator (PCA) on protein expression of metallopeptidase-2 and tissue inhibitor of metalloproteases-2. Data are presented as mean ± standard deviation (n = 8); *P < 0.050 and **P < 0.010 compared with the negative control group; ^#^*P* < 0.050 and ^##^*P* < 0.010 compared with diabetes mellitus group.

Real-time quantitative PCR and Western blotting were used to assess the expression of MMP-2 and TIMP-2. MMP-2 mRNA and protein levels were significantly higher in the diabetes group than in negative controls (*P* < 0.001, Figure 3 and 4); meanwhile, gene and protein TIMP-2 expression was also higher in diabetes group. PCA dramatically ameliorated these changes. These results indicated that PCA decreased the expression of MMP-2 and reduced that of TIMP-2.

## Discussion

In this research, we provided the first evidence that PCA has the potential to attenuate myocardial fibrosis and decrease inflammatory reaction in STZ induced diabetic rats. Based on the obtained results, further investigations were performed by focusing on the potential mechanisms involved. The results indicated that PCA (0.5, 2, and 8 mg/kg) treatment significantly reduced inflammatory lesions, decreased inflammatory cytokine expression, and reduced collagen content and MMP-2 expression. The results suggested that inhibition of myocardial inflammation activation and pro-fibrogenic factor production might be critical mechanism of anti-fibrogenic action of PCA in diabetic rats.

In order to investigate the effects of PCA on myocardial fibrosis in diabetic rats, high-fat diet and intraperitoneal injection of STZ (30 mg/kg) were used to establish a diabetes model. Consistent with previous findings (24), diabetic rats had elevated LVWI, glucose, and myocardial injury, indicating that HF diet and STZ treatment cause significant pathological cardiac remodeling (25). On the other hand, Masson’s trichrome staining indicated a significant increase in the degree of left ventricle myocardial fibrosis in the DM group compared with negative controls (26). However, treatment with PCA (2 and 8 mg/kg) significantly decreased blood glucose level, myocardial fibrosis, and CVF, and these changes were associated with lesion amelioration. Further studies should be carried out to understand the regulatory mechanism of PCA in inducing the blood glucose level in type 2 diabetic rats. We speculated that PCA could stabilize the use of glucose in the peripheral tissues and therefore have an indirect hypoglycemic effect.

Since the inflammation activation plays an important role in cardiac remodeling (27), we assessed whether PCA effects on fibrosis were associated with a modulation of circulating pro-inflammatory cytokine levels. TGF-β1 and IL-1β levels in diabetic rats were found to be elevated, suggesting a sustained inflammation activated by STZ (30 mg/kg) and high-fat diet. Interestingly, PCA treatment obviously decreased the levels of both cytokines. TGF-β1 was shown to trigger inflammatory signaling pathways causing concentric left ventricular hypertrophy and increasing collagen deposition and myocardial fibrosis (28,29). IL-1β was directly correlated with inflammatory reaction, and caused myocardial collagen and cardiomyocyte apoptosis (30,31). Favorable regulatory effects of PCA on TGF-β1 and IL-1β levels suggest that inhibition of these two cytokines might be a key mechanism of PCA anti-fibrotic effects in diabetic rats.

MMPs belong to the family of structurally related zinc containing endopeptidases, which play pivotal roles in inflammatory diseases, including oxidative stress-associated cardiac fibrosis (32). Importantly, increased levels of MMP-2 have been observed in coronary blood samples from patients with myocardial infarction (33). In this study, PCA decreased MMP-2 levels in diabetic rats, suggesting that this protein might control the MMPs balance.

TIMP-2 as endogenous inhibitor plays an important role in the MMP-2 regulation, especially in the development of cardiac dysfunction and myocardial interstitial fibrosis (34). Under normal physiological conditions, MMP-2 forms a non-active complex with its inhibitors, the TIMP proteins. Once the level of cytokines (eg, TGF-β1 and IL-1β) increases, the balance of MMPs and TIMPs is altered with the decomposition of the MMP-TIMP complex. The increase in the activity of MMPs and TIMPs is followed by an increase in the degree of myocardial fibrosis in diabetic rats (35). Our results indicate that PCA not only decreased MMP-2 levels but also sustained the MMP-TIMP balance in the diabetic rat myocardium. These data might partly explain the inhibition of PCA against progressive cardiac dysfunction and myocardial interstitial fibrosis. A limitation of the study may be the diabetic model used, since there are other, more up-to-date type 2 diabetes models, but for the purposes of this study, such models were not of utmost importance.

In conclusion, PCA has potential anti-fibrotic effects, like modulating the balance between inflammatory cytokine levels and collagen content, as well as modulating MMP expression and sustaining the MMP-TIMP balance. These findings indicate that PCA administration could attenuate STZ-induced cardiac fibrosis in diabetic rats and have therapeutic potential against myocardial injury induced by high-fat diet and intraperitoneal STZ administration.
