# Supplementary material for: A new Agkistrodon halys venom-purified protein C activator prevents myocardial fibrosis in diabetic rats
Source: Croat Med J. 2015 Oct;56(5):439–46. doi: 10.3325/cmj.2015.56.439 (PMC4655929; doi:10.3325/cmj.2015.56.439)
Supplement: Supplementary Table 1 [file CroatMedJ_56_s004.pdf]

Reference Scan(s)	Peptide	MH+	z	P (pro) P (pep)	Score XC	Coverage DeltaCn	MW Sp	Accession RSp	Peptide (Hits) Ions	Count
1 acurhagin precursor [Deinagkistrodon acutus]				9,44E-15	90,27		68496,8	45331367	6 (16 0 0 0 0)	
1294	K.DDSPGQNNPCK.M	1231,50083	1	2,30E-03	2,20	0,93	206,3	1	9/20	1
2794	K.DDSPGQNNPCK.M	1231,50083	2	2,46E-07	2,78	0,21	515,7	1	15/20	1
7368	K.HDNAQLITGIDFR.G	1499,76012	2	1,16E-12	4,75	0,85	2269,2	1	22/24	2
4490	K.IPCASEDVK.C	1018,48741	1	9,52E-01	2,03	0,38	476,6	1	11/16	1
9285	K.ITVKPDVDYTLNAFAEWR.K	2138,09168	2	1,22E-14	5,48	0,90	1223,6	1	26/34	3
7059	K.KHDNAQLITGIDFR.G	1627,85508	2	1,72E-13	4,57	0,77	1957,8	1	24/26	2
3708	K.MFYSNDDEHK.G	1285,51538	2	9,26E-09	2,81	0,75	719,4	1	14/18	1
5792	K.VCSNGHCVDVTTAY.	1582,66252	2	2,71E-05	2,80	0,86	767,6	1	17/26	2
6024	R.ASM*SECDPAEHCTGQSSECPADVFHK.N	2953,14928	2	1,84E-05	3,07	0,85	320,8	1	21/50	5
5993	R.ASM*SECDPAEHCTGQSSECPADVFHK.N	2953,14928	3	9,44E-15	4,92	0,91	1185,0	1	36/100	1
7660	R.GSIIGYAYIGSMCHPK.R	1753,84006	2	1,38E-09	3,27	0,85	845,8	1	16/30	2

Coverage - acurhagin precursor [Deinagkistrodon acutus]

Reference: gi|45331367|gb|AAS57937.1| acurhagin precursor [Deinagkistrodon acutus]

Database: D:\Database\Agkistrodon.fasta   Monoisotopic MW: 68497.8   Number of Amino Acids: 610   pI: 4.90

1   305   610

	1-10	11-20	21-30	31-40	41-50	51-60	61-70	71-80	81-90	91-100
1	MIQVLLVTIC	LAAFPYQGSS	IILESGDVND	YEVVYPRKVT	ALPKGAVQQK	YEDAMQYEFK	VNGEPVVLHL	EKNKHLFSKD	YSETHYSPDG	REITINPPVE
101	DHCYYHGRIE	NDGDSTASIS	ACNGLKGNFK	LQGETYLIEP	MKLSDSEAHA	VFKYENVEKE	DEAPKMCGVT	QKWKSYEPIK	KISQLNLIPE	QQIYDPFKYV
201	ETVVVVDKAM	VTKYNGDLDK	IKTKMYEAAN	NMNEMYRYMF	FRVVMVGLII	WTEEDKITVK	PDVDYTLNAF	AEWRKTYLLA	EKKHDNAQLI	TGIDFRGSII
301	GYAYIGSMCH	PKRSVGIIQD	YSPINLVLAV	IMAHEMGHNL	GIHHDDGYCY	CGGYPCIMGP	SISPEPSKFF	SNCSYIQCWD	FIMNHNPECI	DNEPLGTDII
401	SPPLCGNELL	EVGEECDCGT	PENCQNECCD	AATCKLKSGS	QCGHGDCCEQ	CKFRTSGTEC	RASMSECDPA	EHCTGQSSEC	PADVFHKNGE	PCLDNYGYCY
501	NGNCPIMYHQ	CYALFGADIY	EAEDSCFESN	KKGNYYGYCR	KENGKKIPCA	SEDVKCGRLY	CKDDSPGQNN	PCKMFYSNDD	EHKGMVLPGT	KCADGKVCSN
601	GHCVDVTTAY									

Protein Coverage Totals

by Mass:	12985.8
% by Mass:	18.96
by Position:	118
% by AA's:	19.34

Protein Coverage: Include unmatched fragments in table

Sequence	MH+	% by Mass	Position	% by AA's
KVTALPKGAVQQK	1367.84	2.00	38 - 50	2.13
VTALPKGAVQQK	1239.74	1.81	39 - 50	1.97
VTALPKGAVQQKYEDAMQYEFK	2544.28	3.71	39 - 60	3.61
GAVQQK	630.36	0.92	45 - 50	0.98
GAVQQKYEDAMQYEFK	1934.90	2.82	45 - 60	2.62
GAVQQKYEDAMQYEFKVNGEPVVLHLEK	3249.62	4.74	45 - 72	4.59
VNGEPVVLHLEKNKHLFSK	2188.22	3.19	61 - 79	3.11
NKHLFSK	873.49	1.28	73 - 79	1.15
NKHLFSKDYSETHYSPDGR	2281.06	3.33	73 - 91	3.11
HLFSK	631.36	0.92	75 - 79	0.82
HLFSKDYSETHYSPDGR	2038.93	2.98	75 - 91	2.79
EITINPPVEDHCYYHGR	2042.94	2.98	92 - 108	2.79
IENDGDSTASISACNGLK	1794.82	2.62	109 - 126	2.95
IENDGDSTASISACNGLKGNFK	2241.05	3.27	109 - 130	3.61
GNFKLQGETYLIEPMK	1867.96	2.73	127 - 142	2.62
GNFKLQGETYLIEPMKLSDSEAHAVFK	3052.54	4.46	127 - 153	4.43
LQGETYLIEPMK	1421.73	2.08	131 - 142	1.97
LQGETYLIEPMKLSDSEAHAVFK	2606.32	3.80	131 - 153	3.77
LSDSEAHAVFK	1203.60	1.76	143 - 153	1.80
LSDSEAHAVFKYENVEK	1965.96	2.87	143 - 159	2.79
LSDSEAHAVFKYENVEKEDEAPK	2635.25	3.85	143 - 165	3.77
YENVEKEDEAPKMCGVTQK	2198.01	3.21	154 - 172	3.11
EDEAPKMCGVTQK	1435.66	2.10	160 - 172	2.13
EDEAPKMCGVTQKWK	1749.83	2.55	160 - 174	2.46
MCGVTQK	766.36	1.12	166 - 172	1.15
MCGVTQKWK	1080.53	1.58	166 - 174	1.48
MCGVTQKWKSYEPIK	1797.90	2.62	166 - 180	2.46
WKSYEPIK	1050.56	1.53	173 - 180	1.31
WKSYEPIKK	1178.66	1.72	173 - 181	1.48
SYEPIK	736.39	1.08	175 - 180	0.98
SYEPIKK	864.48	1.26	175 - 181	1.15
KISQLNLIPEQQIYDPFK	2174.19	3.17	181 - 198	2.95
ISQLNLIPEQQIYDPFK	2046.09	2.99	182 - 198	2.79
YVETVVVVDK	1150.64	1.68	199 - 208	1.64
YVETVVVVDKAMVTK	1680.92	2.45	199 - 213	2.46
YVETVVVVDKAMVTKYNGDLDK	2486.28	3.63	199 - 220	3.61
AMVTKYNGDLDK	1354.67	1.98	209 - 220	1.97
AMVTKYNGDLDKIK	1595.85	2.33	209 - 222	2.30

Ready   CAP NUM

Start   LS20X-20130328.srf -...   C:\Cluster\pvm\lib\WIN3...   11:39 PM

Score XC	Coverage DeltaCn	MW Sp	Accession RSp	Peptide (Hits) Ions	Count
40,23		16714,9	205830836	6 (6 0 0 0 0)	
2,32	0,72	365,6	1	11/16	4
2,82	0,83	568,3	1	14/16	4
1,98	0,85	863,5	1	12/16	3
2,94	0,82	1156,4	1	14/16	3
4,62	0,59	915,6	1	21/38	1
3,94	0,87	1347,0	1	23/38	1
